# Tg1.4HBV-s-rec mice, a crossbred hepatitis B virus-transgenic model, develop mild hepatitis

**DOI:** 10.1038/s41598-023-50090-8

**Published:** 2023-12-20

**Authors:** Stefan Schefczyk, Xufeng Luo, Yaojie Liang, Mike Hasenberg, Bernd Walkenfort, Martin Trippler, Jonas Schuhenn, Kathrin Sutter, Mengji Lu, Heiner Wedemeyer, Hartmut H. Schmidt, Ruth Broering

**Affiliations:** 1 Department of Gastroenterology, Hepatology and Transplant Medicine, Medical Faculty, University of Duisburg-Essen, Hufelandstr. 55, 45147 Essen, Germany; 2 Institute for Lymphoma Research, The Affiliated Cancer Hospital of Zhengzhou University & Henan Cancer Hospital, Zhengzhou, China; 3 Electron Microscopy Unit, Imaging Center Essen, Medical Faculty, Germany Institute for Virology, University Hospital Essen, University of Duisburg-Essen, Essen, Germany; 4 Institute for Virology, University Hospital Essen, University of Duisburg-Essen, Essen, Germany; 5 Department of Gastroenterology, Hepatology and Endocrinology, Hannover Medical School, Hannover, Germany

**Keywords:** Hepatology, Virology

## Abstract

Hepatitis B virus (HBV)-transgenic mice exhibit competent innate immunity and are therefore an ideal model for considering intrinsic or cell-based mechanisms in HBV pathophysiology. A highly replicative model that has been little used, let alone characterized, is the Tg1.4HBV-s-rec strain derived from cross breeding of HBV-transgenic mouse models that either accumulate (Alb/HBs, Tg[Alb1-HBV]Bri44) or lack (Tg1.4HBV-s-mut) the hepatitis B surface antigen (HBsAg). Tg1.4HBV-s-rec hepatocytes secreted HBsAg, Hepatitis B extracellular antigen (HBeAg) and produced HBV virions. Transmission electron microscopy visualised viral particles (Tg1.4HBV-s-rec), nuclear capsid formations (Tg1.4HBV-s-mut and Tg1.4HBV-s-rec) and endoplasmic reticulum malformations (Alb/HBs). Viral replication in Tg1.4HBV-s-rec and Tg1.4HBV-s-mut differed in HBsAg expression and interestingly in the distribution of HBV core antigen (HBcAg) and HBV × protein. While in Tg1.4HBV-s-mut hepatocytes, the HBcAg was located in the cytoplasm, in Tg1.4HBV-s-rec hepatocytes, the HBcAg appeared in the nuclei, suggesting a more productive replication. Finally, Tg1.4HBV-s-rec mice showed symptoms of mild hepatitis, with reduced liver function and elevated serum transaminases, which appeared to be related to natural killer T cell activation. In conclusion, the study of Alb/HBs, Tg1.4HBV-s-mut and their F1 progeny provides a powerful tool to elucidate HBV pathophysiology, especially in the early HBeAg-positive phases of chronic infection and chronic hepatitis.

## Introduction

Hepatitis B virus (HBV) infection, with approximately 320 million people living with HBV worldwide^1^, is a leading cause of liver-related morbidity and mortality^2^. Progression of chronic HBV infection can be divided into five phases; (I) HBeAg-positive chronic infection, (II) HBeAg-positive chronic hepatitis, (III) HBeAg-negative chronic infection, (IV) HBeAg-negative chronic hepatitis and (V) HBsAg-negative phase^3^. Each phase depends on a distinct pattern of immune activation, although HBV has been described as a stealth virus that evades innate and adaptive immune responses^4,5^. The outcome, chronicity and pathogenesis of HBV infection depend on complex interactions between the immune system and viral proteins.

The host spectrum of HBV is restricted to humans, primates such as *Pan troglodytes* (chimpanzees)^6^ and *Macaca fascicularis* (mauritian cynomolgus monkeys)^7^. *Tupaia belangeri* (tree shrews) have also been reported to be susceptible to HBV infection, but with a transient and mild course^8^. Other members of the *Hepadnaviridae* family, such as duck hepatitis virus and woodchuck hepatitis virus have been used for studies on HBV replication and pathogenesis in their own hosts. However, these host-virus systems are fundamentally different from the interaction of HBV infection in humans^9–11^. Another point is that due to the limited host spectrum, animal models for HBV are not widely engaged^12^. The most commonly used animal models are mouse models. These include HBV-transgenic strains^13,14^, hydrodynamic injection models^12^ and genetically^15^ or liver-humanized^16^ mouse models. All of these models have their advantages and disadvantages. One of the HBV-transgenic models’ setbacks is the natural immune tolerance to HBV antigens, due to self-recognition^17^. However, transgenic mice exhibit competent innate immunity and are therefore an ideal model for considering intrinsic or cell-based innate immune induction and evasion.

In the present study, viral replication was characterized in different HBV-transgenic mouse models. The first model is the Tg[Alb1-HBV]Bri44 (Alb/HBs) strain containing the open reading frame (PreS1 to HBx^18^) of the hepatitis B surface antigen (HBsAg) downstream of the albumin promoter, thus expressing high levels of large, medium and small HBsAg. Originally, it was wrongly assumed that this model showed no signs of pathology^18–20^. However, this model is characterised by persistent inflammation due to the accumulation of HBsAg in the endoplasmic reticulum (ER). This leads to liver damage and ultimately hepatocellular carcinoma (HCC)^19^. The second model, a 1.4 overlength model^14^, shows an endogenous, Kupffer cell (KC)-mediated TLR3 activation^21^. The Tg1.4HBV-S-Mut3 strain (HBV-s-mut) depends on a terminally redundant 1.4 overlength transgene (HBV core to HBx ORF plus terminal redundancy)^14,22^. HBV gene expression and DNA replication are indicated by northern and Southern blot, respectively. This strain replicates HBV but lacks HBsAg expression due to a point mutation from ATG to ACC (T/C position 1438), resulting in the loss of the translational start codon^14^. The third model, an HBsAg-recovered 1.4 overlength model (HBV-s-rec), results from crossbreeding of model one and two^14^. It has been described that this strain has a wild type-like liver phenotype with no inflammation or interferon response^21,23^. Here, liver morphology, viral antigen distribution and disease-related parameters were determined to indicate the influence of chronic viral replication in these distinct models for HBV infection.

## Results

### TEM analyses show normal cell morphology in Tg1.4HBV-s-rec

**Figure 1:**
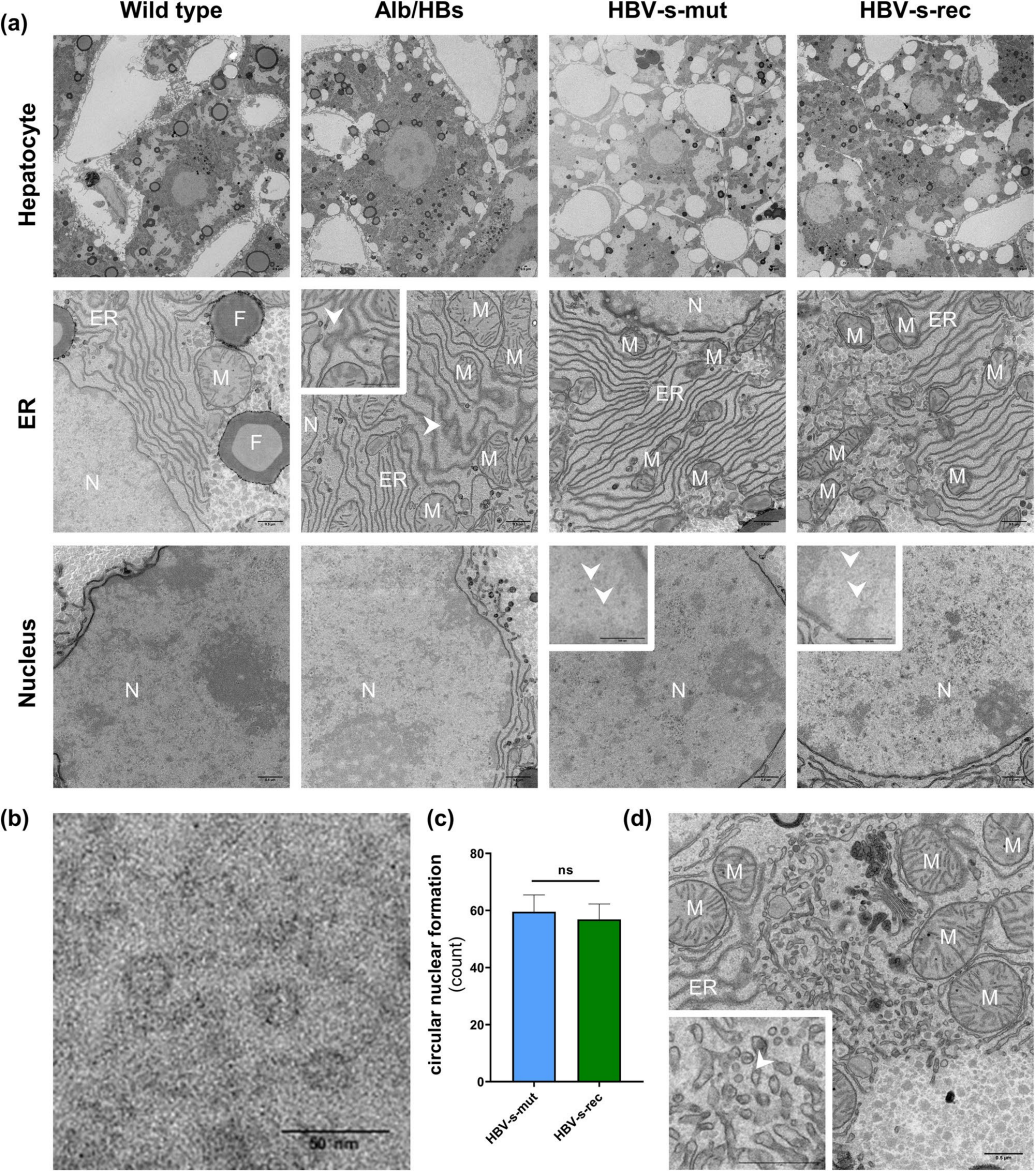
Viral particles are produced in the HBV-s-rec mouse strain. (**a**) Liver tissue from 6-month-old wild type and HBV-transgenic mice were fixated and contrasted according to a modified OTO protocol. Images are representatives of each mouse strain (group sizes *n* = 3). General cell morphology of hepatocytes, the endoplasmic reticulum (ER), cytoplasm, and nuclei are visualised using transmission electron microscopy (TEM). (**b**) Representative high-resolution image of circular structures in a hepatocyte nucleus (HBV-s-mut). (**c**) Quantification of circular nuclear formations in HBV-s-mut and HBV-s-rec (mean ± SD, counts per nucleus, *n* = 10). (**d**) TEM imaging of the Golgi apparatus visualising HBV particle in an HBV-s-rec hepatocyte. M, mitochondria; N, nucleus; F, fat droplets; scale bars, 0.5 µm (**a**, **d**) 50 nm (**b**); nm, nanometer; ns, not significant.

To analyse the impact of transgene-driven HBV protein production and viral replication, TEM was performed to visualise hepatocyte morphology. Liver tissue of 6-month-old HBV-transgenic and wild type mice (group sizes *n* = 3) were analysed. The general morphology of hepatocytes was not affected by transgene expression (Alb/HBs) or virus-driven replication (HBV-s-mut and HBV-s-rec) (Fig. 1a). Focussing on separate intracellular structures, model-specific differences were seen. It is known, that in hepatocytes of Alb/HBs mice the HBsAg accumulates in the ER^20^. Here, TEM indicated ER malformations (arrowhead, ER) in these mice. Malformations were recognised as extensions of the ER structure and were characterised by cross links in the peripheral tubules (Fig. S1). Although, ER structures appeared normal in the HBV-s-mut and HBV-s-rec hepatocytes (Fig. 1a), regions with cross-linked tubular ER could also be seen in the HBV-s-rec strain (Fig. S1). Furthermore, circular structures (arrowheads, Nucleus) were seen in hepatocyte nuclei of HBV-s-mut and HBV-s-rec mice. These spherical structures had a diameter of ~ 20 nm (Fig. 1b). Quantification revealed 60 ± 5 (mean ± SD) and 57 ± 5 in nuclei of HBV-s-mut and HBV-s-rec hepatocytes, respectively (Fig. 1c). Finally, virus-like particles were identified between ER and Golgi apparatus of HBV-s-rec hepatocytes (Fig. 1d, arrowhead).

### Cross-bred Tg1.4HBV-s-rec mice show distinct HBV antigen distribution

**Figure 2:**
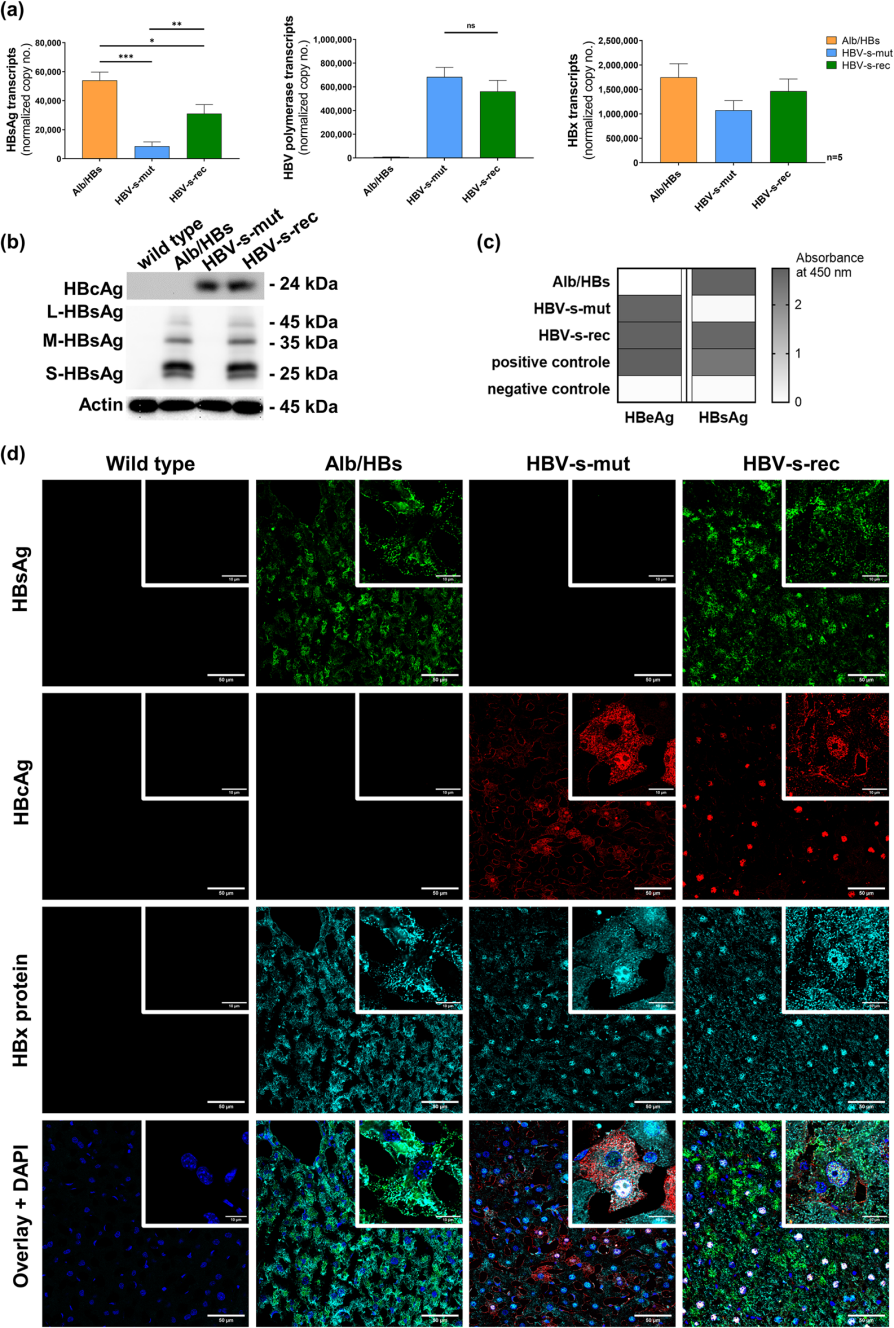
Distinct HBV antigen distribution in Tg1.4HBV-s-rec and its parental mouse strains. (**a**) RNA was extracted from PMH of 3-month-old HBV-transgenic mice (*n* = 5). Gene expression of HBV transcripts (HBsAg, HBV polymerase, and HBx region) was determined by quantitative reverse transcription polymerase chain reaction. Data represent copy numbers as mean ± SD normalized to 100,000 copies of GAPDH. (**b**) Total protein was extracted from liver tissue of 3-month-old wild type and HBV-transgenic mice (*n* = 3) and a western blot analysis was performed to detect HBsAg, HBcAg, and Actin. (**c**) A qualitative ELISA for HBsAg and HBeAg was performed using serum samples of 3-month-old HBV-transgenic mice (*n* = 3). Data are given as heat map of mean values (*n* = 3), representing absorbance at 450 nm. (**d**) Immunofluorescence staining was performed in cryosections of 3-month-old wild type and HBV-transgenic mice to visualise HBsAg, HBcAg and HBx protein. Images are representatives of *n* = 3. Images were acquired using the Leica SP8 gSTED (Leica Microsystems) using a 40 × objective. Scale bars, 50 µm; asterisks indicate significant results *p < 0.05, **p < 0.01, ***p < 0.001; ns, not significant.

Quantitative RT-PCR was performed to determine viral replication, using primer specific for pregenomic RNA/polymerase, HBsAg and HBx regions (Supplementary Table S1). Primer targeting the HBx region, existing in each HBV-coded mRNA, is suggested as marker for viral transcriptional activity. In Alb/HBs and HBV-s-rec mice the HBsAg is highly expressed, with significant maximum expression in the Alb/HBs strain. The HBV-s-mut strain, lacking the promoter activity of the small HBsAg, produced HBsAg transcripts corresponding to PreS1 and PreS2 transcripts. No difference in the expression of the HBx region and the pregenomic RNA/polymerase region was observed, comparing 1.4 overlength HBV-s-mut and HBV-s-rec mice (Fig. 2a). Western blot analysis indicated HBcAg expression in HBV-s-mut and HBV-s-rec mice and expression of HBsAg (small, medium, and large) in Alb/HBs and HBV-s-rec mice, here HBV-s-mut mice remained HBsAg negative (Fig. 2b). Qualitative ELISA indicated the presence of HBsAg in serum of Alb/HBs and HBV-s-rec mice. HBeAg was detectable in the serum of HBV-s-mut and HBV-s-rec mice (Fig. 2c). Immunofluorescent staining indicated equally distributed HBsAg in Alb/HBs and HBV-s-rec liver sections. Interestingly, HBcAg staining showed predominant cytoplasmic versus nuclear location in HBV-s-mut and HBV-s-rec sections, respectively (Fig. 2d). Fluorescence staining for HBx protein showed differences between the HBV-transgenic mice (Fig. 2d). In the Alb/HBs strain, HBx was found uniformly in the cytoplasm of all hepatocytes (Fig. 2d). In contrast, in the HBV-s-mut strain, HBx protein was present in in some nuclei and in the cytoplasm (Fig. 2d). In HBV-s-rec animals, HBx protein was detected in in nuclei and cytoplasm with slightly increased intensity (Fig. 2d). In general, cccDNA is not produced in mice due to a lack of relevant host factors^24^. Therefore, quantitative PCR failed to detect HBV cccDNA in primary hepatocytes of both 1.4 overlength models (Supplementary Table S2). Thus, HBV-s-mut and HBV-s-rec mice differed in I) HBsAg expression, II) HBcAg and HBx protein distribution and III) HBV particle production.

### Tg1.4HBV-s-rec mice produce viral particles

**Figure 3:**
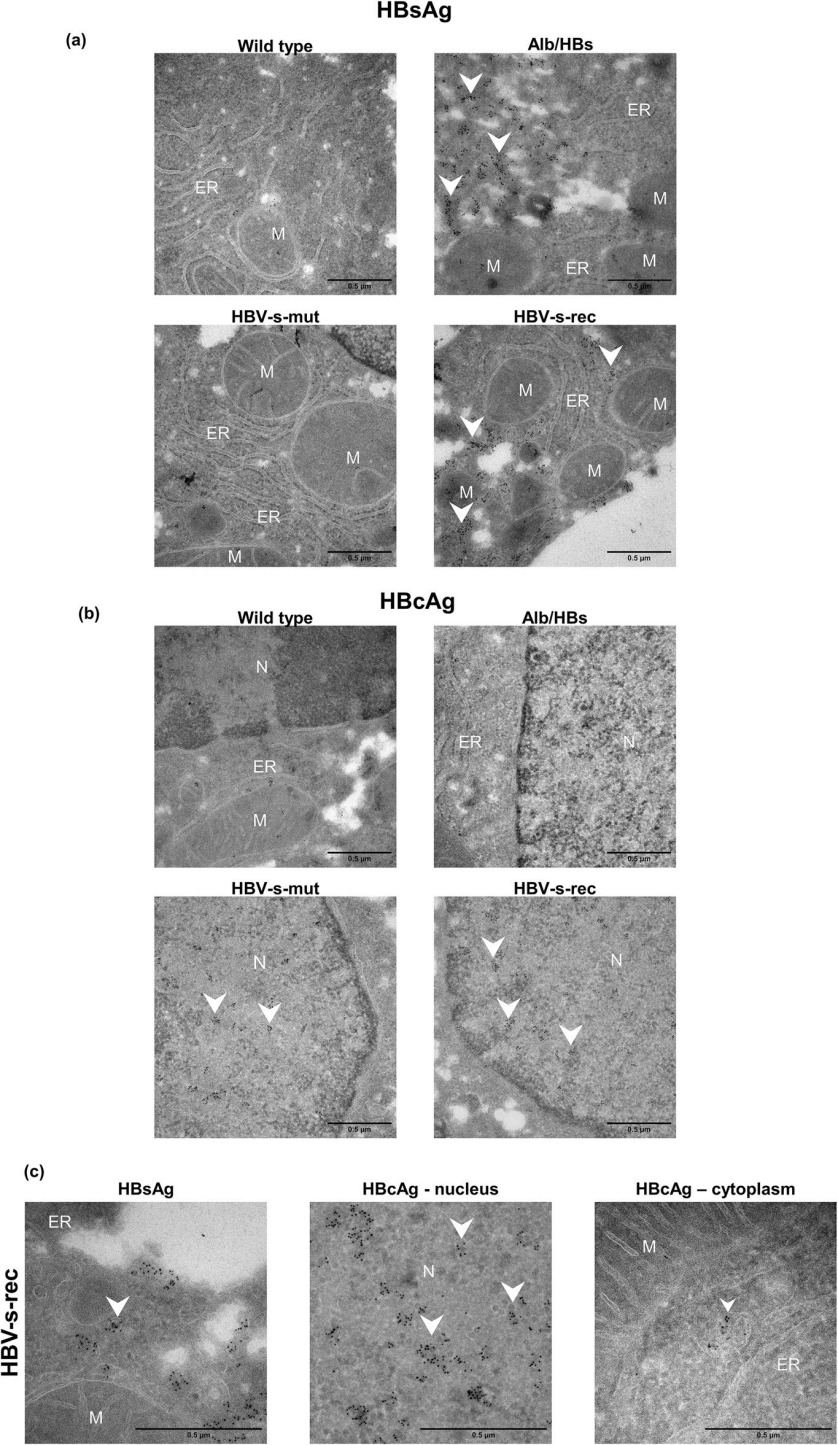
Visualising viral particles in the Tg1.4HBV-s-rec mice. (**a**) Liver tissue from 6-month-old wild type and HBV-transgenic mice (Alb/HBs, HBV-s-mut, HBV-s-rec) were fixated and contrasted according to an immunogold staining protocol. Images are representatives of each mouse strain (group sizes *n* = 3). Endoplasmic reticulum area and nuclei were visualised using transmission electron microscopy. (**a**) Immunogold staining was performed in wild type and HBV-transgenic mouse strains to visualise (**a**) HBsAg and (**b**) HBcAg. (**c**) Representative high-resolution image of viral particle in HBV-s-rec hepatocytes stained for HBsAg and HBcAg. M, mitochondria; N, nucleus; ER, endoplasmic reticulum; scale bars, 0.5 µm.

After investigating the influence of transgene expression (Alb/HBs) and viral driven replication (1.4 overlength models) in the hepatocytes of HBV-transgenic mice, these changes were further analysed by an immunogold staining for HBsAg and HBcAg. The immunogold staining for HBsAg showed that HBsAg was localised at the extensions of the ER and accumulates along these extensions in Alb/HBs mice (Fig. 3a, overview image Fig. S2). Whereas this accumulation along the ER was not found in the HBV-s-rec strain. In this strain the observed viral particles (Fig. 1) were positive for HBsAg (Fig. 3a) and HBcAg (Fig. 3b) after immunogold staining. Despite the similar distribution of HBsAg in the fluorescence staining for confocal microscopy (Fig. 2d), the distribution under the electron microscope looked somewhat different. In the Alb/HBs mice, HBsAg accumulated along the ER, whereas in the HBV-s-rec mice it was mainly found on the viral particles and not at the ER. Despite HBV transcripts given in Fig. 2a indicated a detectable amount of HBsAg in HBV-s-mut mice, HBsAg could not be visualised in these mice by TEM analysis (Fig. 3a) or confocal microscopy (Fig. 2d). The HBcAg staining indicated that the observed circular nuclear formations (Fig. 1b) colocalised with the immunogold staining for HBcAg in HBV-s-mut and HBV-s-rec mice (Fig. 3b). Despite the altered distribution of HBcAg in the HBV-s-mut mice in the fluorescence staining for confocal microscopy (Fig. 2d), this distribution could not be visualised by electron microscopy and immunogold staining. In contrast, the immunogold staining for HBcAg visualised that the viral particles, observed in Fig. 1d were also colocalised to the HBcAg staining in the HBV-s-rec mice (Fig. 3c). In conclusion, TEM showed that transgenic and viral replication has an impact on the ultrastructure of hepatocytes. In particular, HBsAg expression in the Alb/HBs mice led to cross-linked peripheral tubules in the endoplasmic reticulum. Circular nuclear structures in the HBV-s-mut and HBV-s-rec mice, were associated with HBcAg. TEM also showed that HBsAg- and HBcAg-positive particles were assembled along the Golgi apparatus in the HBV-s-rec mice, indicating HBV particles. However, low antibody labelling efficiency and poor epitope preservation during electron microscopy preparation may be responsible for obvious gold-negative ultrastructures that should be positive.

**Figure 4:**
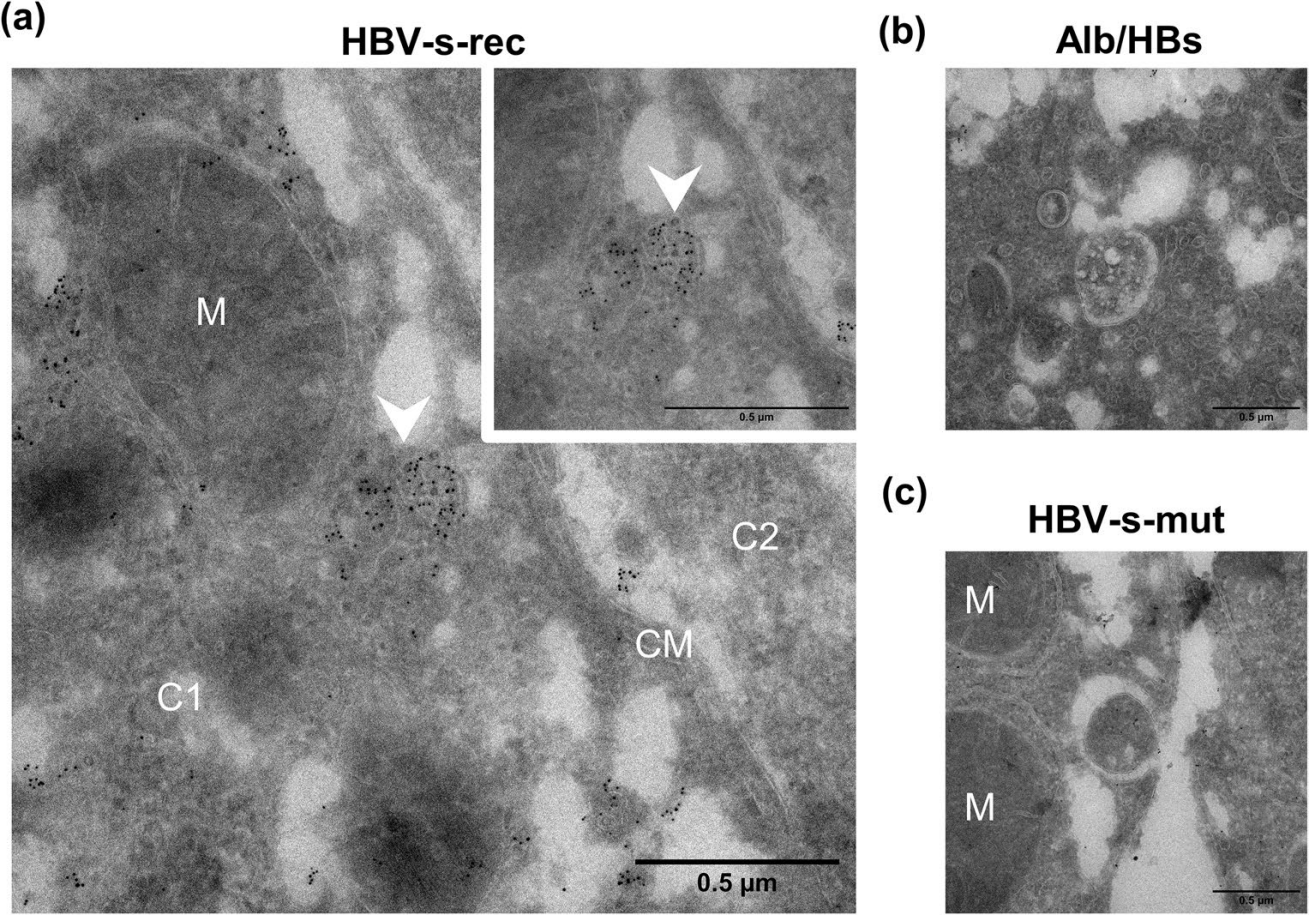
Visualising multivesicular bodies in the Tg1.4HBV-s-rec mice. Liver tissue from 6-month-old HBV-transgenic mice (Alb/HBs, HBV-s-mut, HBV-s-rec) were fixated and contrasted according to an immunogold staining protocol. Images are representatives of each mouse strain (group sizes *n* = 3). Multivesicular bodies were visualised using transmission electron microscopy. Immunogold staining was performed for HBsAg in HBV-s-rec (**a**) and Alb/HBs (**b**) and for HBcAg in HBV-s-mut (**c**) liver tissue. M, mitochondria; C1, cell 1; C2 cell two; CM, cell membrane.

HBV uses different cellular trafficking machinery to assemble and release its particle^25^, TEM micrographs visualised HBV particles between the ER and Golgi apparatus, suggesting that the trans-golgi network may be involved in HBV particle release in HBV-s-rec mice. It is widely accepted that HBV particles are also released through multivesicular bodies (MVBs)^25^. Therefore, TEM images of immunogold staining for HBsAg (Alb/HBs, HBV-s-rec) and HBcAg (HBV-s-mut) were taken to visualise MVB. In the HBV-s-rec mice, accumulation of HBsAg-positive particles in MVB was observed, which were located near the apical plasma membrane (Fig. 4a). The MVB observed in the Alb/HBs (Fig. 4b) and HBV-s-mut (Fig. 4c) strains were immunogold-negative. These findings suggest that HBV particle release also occurs via the MVB path in HBV-s-rec mice.

### Tg1.4HBV-s-rec mice exhibits symptoms of mild hepatitis

To further investigate the consequences of viral and transgenic HBV replication, serum analysis of 3-month-old wild type, Alb/HBs, HBV-s-mut and HBV-s-rec mice (group sizes *n* = 3) was performed using the Spotchem™II liver-1 kit. Here, the HBV-s-rec mice showed significantly decreased albumin and total protein levels, indicating decreased liver function. Additionally, AST/GOT was significantly increased, while ALT/GPT levels remained normal (Table 1), although tending to be increased in Alb/HBs mice. Hematoxylin and eosin staining revealed infiltrating immune cells in inflammatory Alb/HBs liver tissue, whereas mild hepatitis in HBV-s-rec livers did not coincide with immune cell infiltration (Fig. S3). These data for the first time showed that HBV-particle production in HBV-s-rec mice interestingly associated with mild hepatitis, represented by liver damage and suppressed liver function.

**Table 1:** Spotchem™ II liver panel measured in serum of wild type and HBV-transgenic mice.

	Wild type	Alb/HBs	HBV-s-mut	HBV-s-rec
	Mean ± SD	Mean ± SD	Mean ± SD	Mean ± SD
Total protein (g/dl)	5.37 ± 0.17	4.93 ± 0.21	5.13 ± 0.75	3.63* ± 0.73
Albumin (g/dl)	2.50 ± 0.08	2.33 ± 0.05	2.43 ± 0.19	1.47* ± 0.34
Total Bilirubin (mg/dl)	1.03 ± 0.21	0.70 ± 0.43	1.37 ± 0.95	0.83 ± 0.42
ALT/GPT (IU/I)	160.00 ± 102.81	237.00 ± 25.31	147.67 ± 102.85	161.33 ± 67.73
LDH (IU/I)	2895.67 ± 649.27	2270.00 ± 629.69	2,654.67 ± 960.79	3298.67 ± 991.84
AST/GOT (IU/I)	285.33 ± 85.39	237.33 ± 90.71	331.00 ± 133.01	883.67* ± 164.52

Asterisks indicate significant results (*p < 0.05); group sizes *n* = 3; GPT/ALT, alanine aminotransferase.

*LDH* lactate dehydrogenase, *GOT/AST* aspartate aminotransferase, *IU* international units, *SD* standard deviation.

### Hepatic NK and NKT cell populations are increased in Tg1.4HBV-s-rec mice

**Figure 5:**
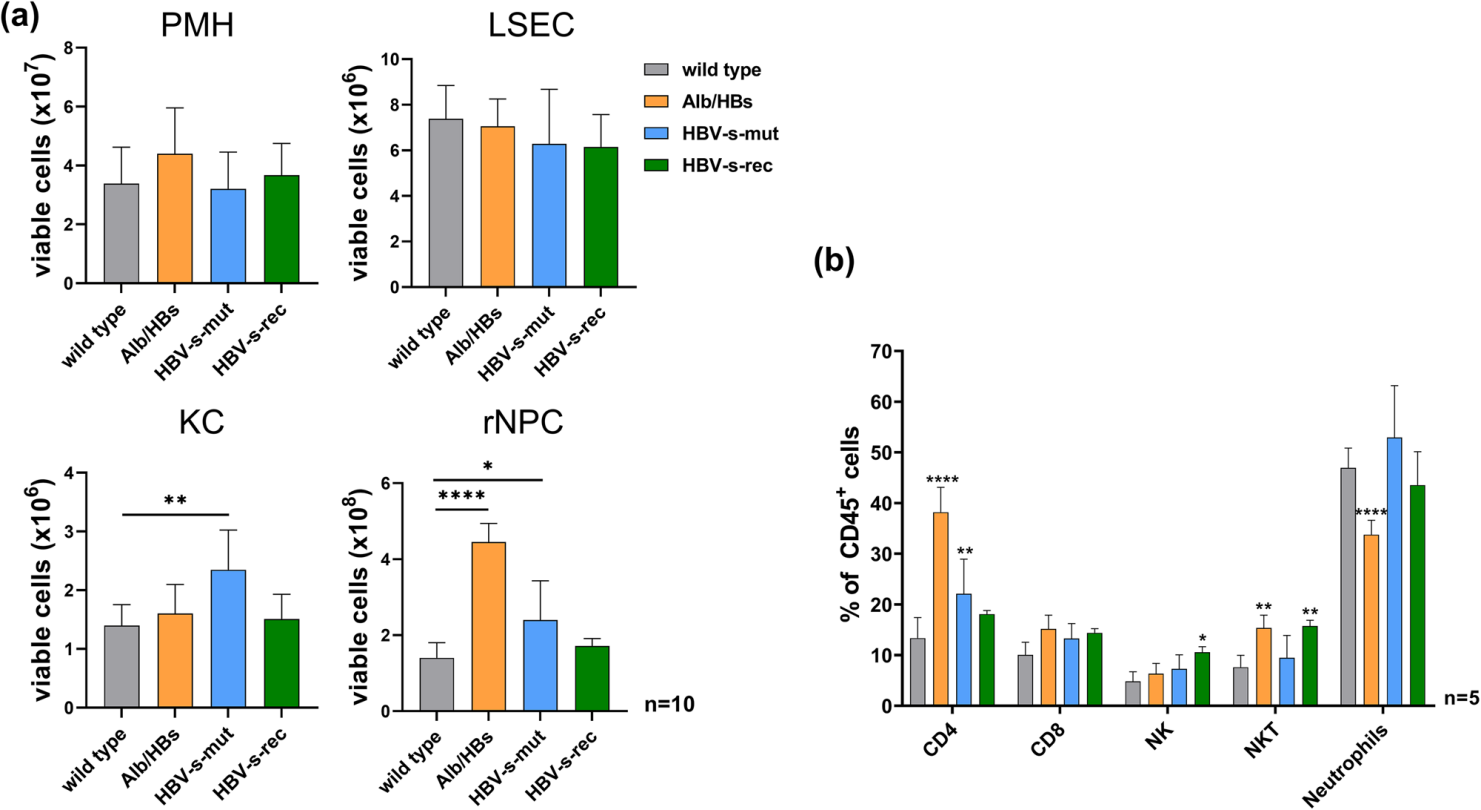
Distinct non-parenchymal liver cell composition in HBV-transgenic mouse strains. (**a**) All-in-one liver cell preparation was performed with 3-month-old wild type and HBV-transgenic mice to obtain PMH, LSEC, KC, and rNPC. Viable cells were counted (*n* = 10) using the Vi-CELL™ XR Cell viability analyser. (**b**) 100,000 rNPC (*n* = 3) were further analysed by flow cytometry, using marker for CD45, CD4, CD8, NK, NKT cells, and neutrophils. Data represent mean % value ± SD of CD45^+^ rNPC. KC, Kupffer cells; LSEC, liver sinusoidal endothelial cells; NK, natural killer cells; NKT, natural killer T-cells; PMH, primary mouse hepatocytes; rNPC, remaining non-parenchymal cells. Asterisks indicate significant results *p < 0.05; **p < 0.01; ***p < 0.001; ****p < 0.0001.

All-in-one liver cell preparation was performed, and yields of PMH, LSEC, KC, and rNPC populations were quantified (*n* = 10) using the Beckman Coulter Vi-Cell™ XR analyser (Fig. 5a). According to KC pre-activation in HBV-s-mut mice^21^, KC counts were significant higher in HBV-s-mut than in the other strains. The rNPC number was significantly increased in Alb/HBs and HBV-s-mut mice, compared to the wild type control (Fig. 5a). Fractions of rNPC were further differentiated using flow cytometry. CD45^+^ cells were analysed and differentiated into CD3^+^ and CD4^+^, CD8a^+^, NK1.1^+^, or CD154^+^ populations, as well as CD3^−^, and NK1.1^+^ and Ly6G^+^ populations (Fig. S4). Compared to wild type mice, no difference in hepatic CD8a^+^ T cell numbers was seen in HBV-transgenic mouse strains. The amount of CD4^+^ T cells was significantly increased in Alb/HBs and HBV-s-mut mice. In the HBV-s-rec strain the amounts of NK and NKT cells were significant higher compared to the wild type control. The NKT cell population in the Alb/HBs strain was also increased, whereas the HBV-s-mut strain showed no differences in these populations compared to the wild type control. The neutrophil population was significantly decreased in the Alb/HBs strain, here no differences were seen between wild type, HBV-s-mut, and HBV-s-rec mice (Fig. 5b). Analysis of the different cell populations revealed a distinct distribution of immune cells within HBV-transgenic strains, however hematoxylin and eosin staining failed to indicate immune cell infiltration (data not shown).

**Figure 6:**
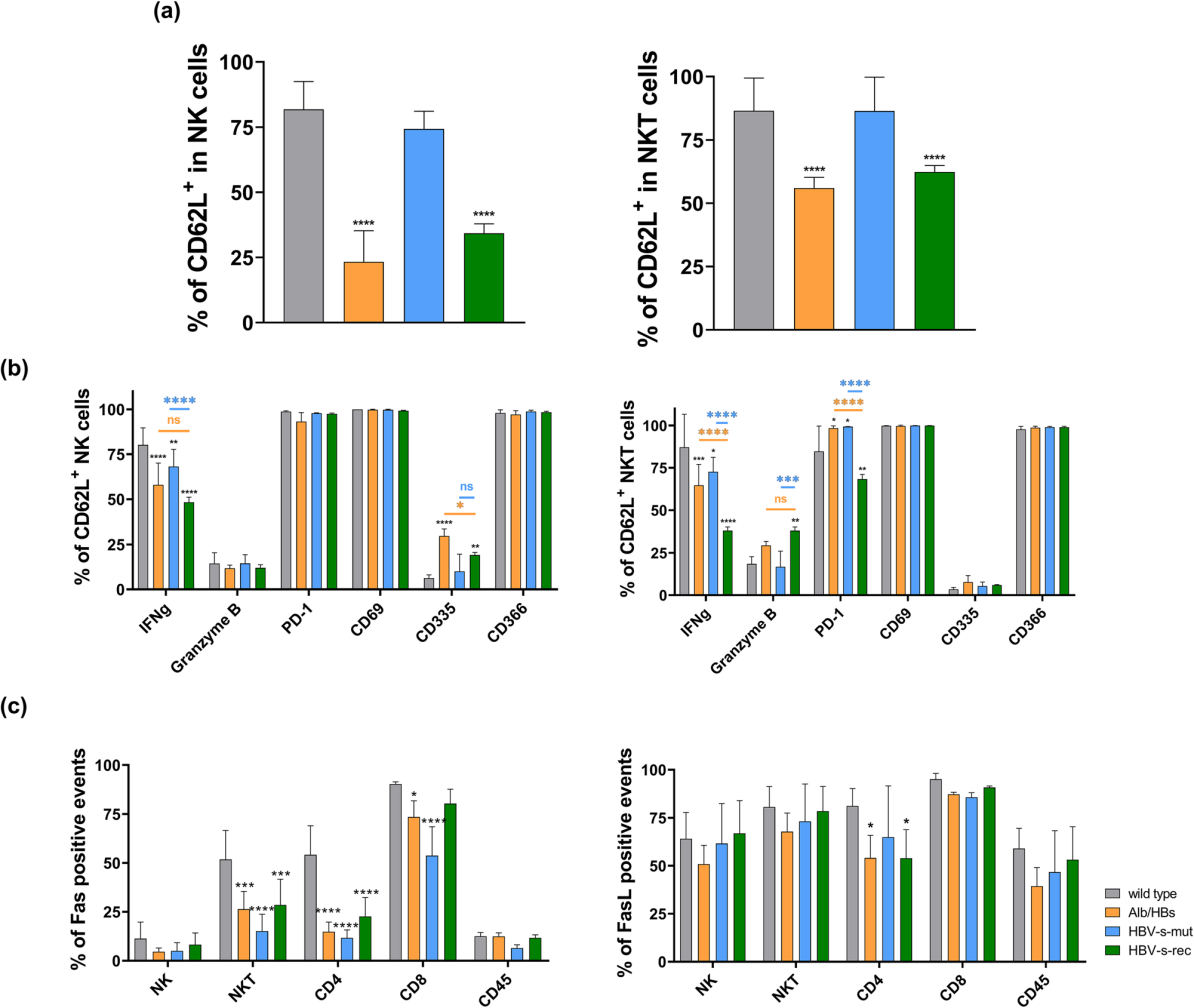
NK and NKT cells show distinct activation in HBV-transgenic mouse strains. Flow cytometry was used to characterise NK and NKT cells. Following an all-in-one liver cell preparation 500,000 rNPC (*n* = 3) were further characterised using markers for CD45, CD3, NK1.1, CD62L, CD69, CD335, CD366, PD-1, granzyme B and IFNg. (**a**) After gating the cells of interest and doublet exclusion (Supplemental Fig. 5), CD45^+^ and NK1.1^+^ cells (NK cells) and CD45^+^, CD3^+^ and NK1.1^+^ cells (NKT cells) were differentiated based on the maturation marker CD62L. (**b**) The CD62L^+^ NK/NKT populations were further analysed for CD69, CD335, CD366, PD-1, granzyme B and IFNg. (**c**) Fas and FasL frequencies were determined in cell populations of interest. Data represent mean % value ± SD of input cells. NK, natural killer cells; NKT, natural killer T-cells; rNPC, remaining non-parenchymal cells. Asterisks indicate significant results *p < 0.05; **p < 0.01; ***p < 0.001; ****p < 0.0001.

Due to the increase in the NK cell population in the HBV-s-rec strain and the increased NKT cell population in the Alb/HBs and HBV-s-rec mice, these populations were further differentiated (Fig. S5). Initially, NK and NKT cells were distinguished based on the differentiation marker CD62L^26^. In total, the number of mature NK and NKT cells were significantly reduced in Alb/HBs and HBV-s-rec mice, when compared to the wild type and HBV-s-mut strains (Fig. 6a). Activation of mature NK and NKT populations were further differentiated for IFNg, granzyme B, PD-1, CD69, CD335 and CD366 (Fig. 6b). The basic activation marker CD69^27^ and the inhibitory marker CD366^28^ did not differ in NK and NKT populations between wild type and HBV-transgenic mice. CD335 is a marker indicating the cytotoxic activity of NK and NKT cells^29^. The NKT cells in all mouse strains showed no variation, but in the Alb/HBs and HBV-s-rec strain, there was an increased expression of CD335 in the NK cells. The antiviral secretory factor IFNg^30^ was reduced in HBV-transgenic NK and NKT cells. Finally, there was an increased level of granzyme B in the matured NKT cells of the HBV-s-rec strain. The inhibitory PD-1^31^ differed in the NKT cells. While PD-1 appeared to be slightly increased in the Alb/HBs and HBV-s-mut strains compared to the wild type, it was significantly decreased in the HBV-s-rec strain. In HBV-transgenic mice, NK and NKT cell populations showed distinct activation pattern. As no differences were seen in NK cell characterisation, when comparing Alb/HBs and HBV-s-rec mice, it can be suggested that the increasing number of granzyme B^+^/PD-1^−^ NKT cells contribute to mild hepatitis progression in HBV-s-rec mice.

The interaction between Fas ligands (FASL) and receptors (FAS) plays an important role in immune regulation. FASL is a type II transmembrane protein expressed on cytotoxic T lymphocytes and natural killer (NK) cells. When it binds to FAS, this leads to programmed cell death. Flow cytometry for FAS and FASL was assessed in NK, NKT, CD4^+^, CD8^+^ and residual CD45^+^ cell fractions. The results indicate a decrease in the frequency of FAS^+^ NKT and CD4^+^ cells in all HBV transgenic models. In addition, the Alb/HBs and HBV-s-mut strain showed a reduced number of FAS^+^ CD8^+^ cells. Conversely, the FAS^+^ CD8^+^ cells of the HBV-s-rec strain were comparable to those of the wild type strain (Fig. 6c). For the remaining CD45^+^ cells, there was no difference in FAS^+^ cells between the wild type and HBV-transgenic strains. In contrast, FASL^+^ CD4^+^ cells were reduced in the Alb/HBs and HBV-s-rec strains compared to the wild type and HBV-s-mut strains. Interestingly, there were no distinguishable levels of FASL^+^ in NK, NKT, CD8^+^ and other CD45^+^ cells. Therefore, Fas ligand and receptor interactions do not appear to provide an explanation for mild hepatitis symptoms in the HBV-s-rec strain.

## Discussion

The HBV-s-rec mouse model, including the parental strains, allows a deeper insight into the different HBsAg-related hepatic immune phenotypes. This HBV-s-rec strain, capable of viral replication, antigen secretion and HBV particle production was created by complementation of two HBV transgenes (HBV-s-mut × Alb/HBs). In contrast to the commonly used HBV-transgenic 1.3 overlength model, which lacks cytopathic effectors and thus induction of hepatitis symptoms^13,32^, the present HBV-s-rec model showed reduced liver function, elevated serum transaminases and NKT effector functions. Our study described the HBV-s-rec strain as a valuable mouse model for studying the viral life cycle to gain insight into HBV-related, immune-mediated pathophysiology.

Solely expressed HBsAg accumulates in the endoplasmic reticulum of hepatocytes and is not released. This accumulation and subsequent ER stress will damage the hepatocyte over time and cause persistent inflammation followed by HCC progression^18–20^. TEM visualized the changes in the ER of Alb/HBs, and immunogold staining confirmed that the change was associated with HBsAg. TEM and confocal microscopy showed that HBsAg was also found along the ER in the HBV-s-rec strain. Here, viral particles were formed and transported via the Golgi apparatus. Thus, no pathologic HBsAg accumulation and thus hepatocyte damage occurred in these animals. Interestingly, the HBV-s-mut strain showed no evidence of HBsAg, even though the point mutation only eliminates small HBsAg but not medium and large HBsAg^21,33^. HBcAg can assemble into icosahedral particles under certain charged conditions^34,35^. These intracellular particles are capable of movement between the nucleus and the cytoplasm^36^. The localisation of HBcAg is of clinical relevance. Whereas patients in whom HBcAg is predominantly located in the nucleus have a mild form of hepatitis, patients with active hepatitis have HBcAg that is predominantly located in the cytoplasm^35,37–39^. It is interesting to note that the confocal images showed that HBcAg is present in both the nucleus and the cytoplasm in the HBV-s-mut strain, whereas it is predominantly located in the nucleus in the HBV-s-rec strain. It should also be pointed out that HBcAg could not be detected in the cytoplasm of the HBV-s-mut strain by immunogold staining using TEM. It should be noted that confocal microscopy has a greater three-dimensional depth, whereas TEM can only examine a small part of a cell in cross-section.

It is considered that CD4^+^ T cells contribute to anti-viral and anti-tumour immune response by producing cytokines that lead to activation of CD8^+^ T cells^40^. Thus, transgene-driven replication and release of HBsAg and HBeAg can lead to an increased number of CD4^+^ T cells. Here, despite the release of HBsAg and HBeAg in the HBV-s-rec mice, the T cell populations were similar to the wild type mice. In the HBV-transgenic mice, the self-recognition by the adaptive immune system likely leads to a lack of specific CD4^+^ and CD8^+^ T cell response to HBV antigens. The rNPC represent about 25% of the total liver cells^41^, herein the populations of T cells, NK, and NKT cells represent the largest proportion. Under healthy conditions, the numbers of CD4^+^ and CD8^+^ tend to be more equalised, but this is not the case in the Alb/HBs and HBV-s-mut mice. Due to their antiviral activity, neutrophils provide further damage to liver tissue in persistent inflammation^42^. This might lead to an acceleration of liver damage in the Alb/HBs strain. Interestingly, despite the ongoing inflammation in the Alb/HBs mice, the population of neutrophils was reduced compared to the wild type. Furthermore, NK and NKT cells have important effector functions in innate immunity^43–45^. These cytotoxic cells can directly lyse infected cells and release pro-inflammatory cytokines. The difference between NK and NKT cells is the lack of a T cell receptor in NK cells^43^, thus NKT cells play a role in both innate and adaptive immune responses. In chronic HBV-infected patients, NK cells possess a phenomenon called "functional dichotomy" which is manifested by a reduced release of pro-inflammatory cytokines but an increase in cytotoxic activity^46^. It might be suggested that the increased number of NK and NKT cells in the HBV-s-rec strain might be involved in the observed liver damage. Nevertheless, the increased number of NK and NKT cells showed that despite HBsAg dominant immune evasion, a local immune response in combination with liver damage occurred in HBV-s-rec mice. Although NK and NKT cell numbers were increased in the HBV-s-rec strain, the frequency of differentiated, CD62L + NK and NKT cells was significantly lower in Alb/HBs and HBV-s-rec mice. However, cytopathic CD335 + NK and granzyme B + /PD-1- NKT cells might be responsible for the hepatitis symptoms in HBV-s-rec mice.

The production and release of HBsAg and viral particles caused liver damage in HBV-s-rec mice that is possibly mediated by a local NKT cell response. In perspective, detailed effector functions of NK, NKT and KC that may underlie the distinct pattern of liver injury in the present mouse models remain to be elucidated. To overcome the limitations of adaptive immunity, associated with HBV-transgenic mice, immune cell transfer might be considered. In summary, the HBV-s-rec strain has the potential to be used as a model to provide deeper insights into host virus interactions in HBeAg-positive chronic hepatitis and may therefore contribute to improving therapeutic interventions.

## Materials and methods

### Animals

Tg(Alb1-HBV)Bri44 mice highly express large, medium, and small HBsAg, designated as Alb/HBs^20^. Tg1.4HBV-S-Mut3 mice harbour an HBV 1.4 overlength genome including a point mutation from ATG to ACG, eliminating the translational start codon of the small HBsAg gene, referred to as HBV-s-mut^14^. Crossbreeding of these two strains (Tg1.4HBV-s-mut × Alb/HBs) generates HBV-transgenic mice, exhibiting a recovered HBsAg expression, described as Tg1.4HBV-s-rec^21^, here named HBV-s-rec. HBV-transgenic strains are on C57BL/6 background. HBV-negative littermates were used as controls, referred to as wild type mice. Mice were bred at the University Hospital of Essen, fed ad libitum and received humane care according to the criteria outlined in the ARRIVE guidelines, in accordance with the U.K. Animals (Scientific Procedures) Act, 1986 and associated guidelines, EU Directive 2010/63/EU for animal experiments. Animal breeding was approved by the local committee (*Landesamt für Natur, Umwelt und Verbraucherschutz,* LANUV Az. 81-02.04.2019.A344).

### Sample preparation for transmission electron microscopy

For transmission electron microscopy (TEM) analyses, organs were primarily perfusion-fixed in situ, immediately after culling, using 5 ml of fixative (4% formaldehyde/2.5% glutaraldehyde in 0.1 M PHEM buffer (60 mM PIPES, 25 mM HEPES, 10 mM EGTA, and 2 mM MgCl_2_)). After hepatectomy, livers were immediately submerged into fixative, in which the organs were trimmed to cubes of 3 mm edge length using a scalpel. Tissue cubes were incubated for 60 min at RT in the fixative. A final aldehyde fixation step was performed by use of a laboratory microwave oven (PELCO BioWave Pro+, Ted Pella, Redding, USA). The tissue cubes, floating in fixative, were exposed four times to microwave radiation (100 W, 2 min, 20 °C steady temp, under vacuum conditions) interrupted by a cooling step (0 W, 2 min, 20 °C steady temperature, under vacuum conditions). Subsequently, the samples were processed according to a modified OTO (osmium-thiocarbohydrazide-osmium) protocol^47^, details are given as supplemental information (Supplementary Table S3). After resin infiltration, the cubes were placed in flat silicone molds, and the resin was polymerized at 60 °C for 96 h. For TEM analysis, ultra-thin sections with a diameter of 55 nm were cut using an ultramicrotome (EM UC7, Leica Microsystems, Wetzlar, Germany) and transferred to 200 mesh hexagonal thin-bar TEM copper grids (Science Services, Munich, Germany).

### Sample preparation for immunoelectron microscopy

Immunogold analysis was adapted from the Tokuyasu cryosectioning protocol^48,49^: Tissue biopsy cubes were prepared as described above for standard TEM, except that the fixative solution was changed to 2% FA + 0.2% GA in 0.1 M Sorensen's phosphate buffer (PB; 0.1 M Na_2_HPO4 + 0.1 M NaH_2_PO_4_ dissolved in deionised water, pH 7.3). Fixation was carried out on a rotary incubator for 3 h before the cubes were washed 3 times for 5 min each with pure PB. To reduce false antibody binding by free aldehyde groups at a later stage, these were blocked by incubating the tissue cubes in 0.15% (w/v) glycine dissolved in phosphate-buffered saline (PBS; 137 mmol NaCl, 2.7 mmol KCl, 10 mmol Na_2_HPO_4_, NaH_2_PO_4_) for 10 min on a rotary incubator. The cubes were then transferred to a gelatin solution (12% gelatin (Rousselot 250, Lime Bone Pork (LP) 30) dissolved in 0.1 M PB + 0.02% sodium azide) prewarmed to 37 °C and incubated at 37 °C for 30 min. The biopsy cubes were then firmly embedded in the gelatine by placing them, together with an appropriate amount of adherent gelatine, between two microscope slides for 30 min on ice. Tissue cubes of 1 mm edge length were cut from the resulting thin gelatin layer and immediately transferred to a sucrose solution (2.3 M in PB), where they remained overnight at 4 °C on a rotary incubator. The cubes were then frozen on cryo-pins with liquid nitrogen and ultrathin cryosections (300 µm × 300 µm × 50 nm) were prepared using a cryo-ultramicrotome (EM UC7 with FC7, Leica Microsystems) with a 45° cryo-diamond knife (Diatome, Hatfield, USA). After ultramicrotomy, sections were transferred to 200 mesh Formvar/carbon-coated copper grids (Science Services, Munich, Germany) using a 1:1 mixture of 2.3 M sucrose in PB and 2% methylcellulose (MC) in deionised water. These samples were stored at 4 °C for several weeks until immunostaining. The following antibodies were used for immunostaining as described in Supplementary Table S4. HBV core antigen was detected using the primary anti-HBcAg antibody (Abcam, Cambridge, UK, mouse IgG2a isotype; final antibody concentration of 10 µg/ml) together with a secondary 6 nm gold nanoparticle-coupled goat anti-mouse IgG (H&L) antibody (Aurion, Wageningen, The Netherlands; final dilution of commercial antibody solution of 1:20). The HBV surface antigen was labelled using a primary anti-HBsAg antibody (Aviva System Biology, San Diego, USA, rabbit IgG isotype; final concentration 10 µg/ml) together with a secondary 6 nm gold nanoparticle coupled goat anti-rabbit IgG (H&L) antibody (Aurion; final dilution of commercial antibody solution 1:20). The antibody labelling protocol started with a 30 min incubation of the respective sample grids on pure PBS at 37 °C to remove adherent gelatine. All subsequent incubation steps were performed using a grid-on-drop approach with a drop volume of 35 µl at room temperature (RT). The samples were first blocked with 0.15% (w/v) glycine dissolved in PBS (3 × 2 min each), followed by 1% (w/v) bovine serum albumin (BSA), and dissolved in PBS (1 × 5 min). The grids were then incubated overnight on drops of primary antibody solution diluted to final concentration with 1% (w/v) BSA in PBS. After washing (5 × 2 min each) with 0.1% BSA in PBS, the samples were incubated on secondary antibody solutions diluted to final concentration with 1% (w/v) BSA in PBS. After further washing (5 × 2 min each) with 0.1% BSA in PBS, the samples were post-fixed with 1% GA in PBS for 5 min. Subsequent washes (6 × 1 min) were performed with deionised water followed by contrasting with 2% uranyl acetate (UA) dissolved in 0.15 M oxalate at pH 7 for 10 min. After a short wash step with deionised water (10 s) at RT, the grids were placed on ice for 10 s on drops of a 1:10 mixture (pH 4) of 4% UA and 2% MC, both dissolved in deionised water. After transferring the grids to fresh drops of the UA/MC mixture, an incubation step was performed for 10 min, again on ice. Finally, the grids were ‘looped out’ of the UA/MC drop using a remanium wire loop, the excess UA/MC was removed with a filter paper and the samples were air dried for 30 min. The grids were then stored at RT until TEM observation in the range of one week.

### Transmission electron microscopy

Image acquisition was performed with a JEM 1400Plus TEM (JEOL Ltd., Tokyo, Japan), operating at 120 kV and equipped with a 4096 × 4096 pixels CMOS camera (TemCam-F416, TVIPS, Gauting, Germany). Resulting 16-bit TIFF images were processed using either Adobe® Photoshop® CS4 or Fiji (v1.53c)^50^.

### Spotchem™II analysis and enzyme linked immunosorbent assay

Serum analyses of alanine aminotransferase (ALT/GPT), aspartate aminotransferase (AST/GOT), albumin, lactate dehydrogenase (LDH), total bilirubin, and total protein were performed for 9-week-old wild type and HBV-transgenic mice (*n* = 3) with the Spotchem™II analyser and Spotchem™II reagent strips (Liver-1 panel; Arkray, Kyozo, Japan) as previously described^21^. HBsAg (Novus Biologicals, Littleton, USA) and hepatitis B extracellular antigen (HBeAg; Aviva Systems Biology, San Diego USA) were detected in serum samples of HBV-transgenic mice (*n* = 3) using qualitative enzyme linked immunosorbent assays (ELISA), analysed with FluoStar™ microplate reader, according to manufacturer´s instructions.

### All-in-one liver cell preparation

Primary mouse hepatocytes (PMH), liver sinusoidal endothelial cells (LSEC), and KC were prepared from 6 to 9-week-old wild type and HBV-transgenic mice (Alb/HBs, HBV-s-mut, and HBV-s-rec) by a two-step in situ perfusion as previously described^51^. After bead-based depletion of LSEC and KC the final fraction was used as remaining non-parenchymal cells (rNPC).

### RNA isolation and one-step quantitative reverse transcription polymerase chain reaction

Total RNA was isolated and purified using Qiazol™ solution (Qiagen, Hilden, Germany) and RNeasy Mini Kit (Qiagen) according to the manufacturer's instructions. Quantitative reverse transcription polymerase chain reaction (RT-PCR) was performed using the QuantiFast SYBR Green RT-PCR Kit (Qiagen). Primers are listed in Supplementary Table S1.

### Western blot

Total protein lysates of PMH from wild type and HBV-transgenic mice (*n* = 3) were prepared using RIPA buffer (50 mM Tris/HCl (pH 7.4), 150 mM NaCl, 1 mM NaF, 1 mM EDTA, 0.1% SDS (w/v), 1% NP40 (v/v), 0.5% Na-Deoxycholat (w/v), protease and phosphatase inhibitors (Roche, Basel, CHE)). Lysates were centrifuged for 20 min at 14,000×*g* and 4 °C. Samples were prepared in 8 × loading buffer and incubated at 98 °C for 5 min. Western blots were performed as described elsewhere^52^. Antibodies are listed in Supplementary Table S5.

### Immunocytochemical staining

Standard cytochemical staining protocols (different manufacturers) were applied to liver cryosections from wild type and HBV-transgenic mice (n = 3). Organs were perfusion-fixed in situ using 4% paraformaldehyde in PBS and fixed overnight. Tissues were equilibrated in 30% sucrose for 24 h and frozen in Tissue-Tek OCT (Sakura Finetek GmbH, Alphen aan den Rijn, Netherlands). Sectioning was performed at − 25 °C by using a cryostat (Leica Microsystems) with a thickness of 7 µm. Sections were rehydrated in PBS for 10 min and blocked in 1% BSA and 5% natural donkey serum. Primary antibodies against HBsAg, Hepatitis B core antigen (HBcAg) and HBx protein (HBx) were used and visualized using fluorophore-conjugated secondary antibodies (Supplementary Table S6). Primary antibody incubation was performed at 4 °C overnight in blocking solution (Table S6). After washing secondary antibody staining was performed for one hour at room temperature in blocking solution (Table S5). Sections were washed with PBS and mounted using Fluorshield™ with DAPI (Merck Millipore, Burlington, USA). Image acquisition was performed with a Leica SP8 gSTED (Leica Microsystems) using a 40 × objective and further processed using Imaris (Oxford Instruments, Abingdon, United Kingdom).

### Hematoxylin and eosin staining

Tissue sections were prepared as described for immunofluorescence staining. Fixed cryosections were washed twice with PBS for 5 min at room temperature to remove the OCT and briefly washed with water. Sections were incubated in hematoxylin (Carl Roth, Karlsruhe, Germany) for 6 min and washed in a stream of tap water for 6 min. Eosin (Carl Roth, 1% [w/v]) staining followed for 5 s, sections were directly dehydrated in an increasing ethanol series (70% and 90%) by briefly dipping the coverslip into the solution. Finally, sections were dipped in an isopropanol and afterwards xylol solution before mounted with Canada balsam (Sigma-Aldrich) for long-term preservation.

### Flow cytometry

Remaining NPC were further analysed (*n* = 3) using flow cytometry (BioLegend® standard protocol). Here, 100,000 cells were resuspended in PBS (containing 1% FCS and 20 mM EDTA) and antibody staining (Supplementary Table S7) was performed for 30 min at room temperature. Cells were centrifuged at 300×*g* for 5 min at 4 °C, washed with PBS containing 1% FCS and 20 mM EDTA and measured using CytoFLEX S (Beckmann Coulter, Brea, CA US) and analysed using FlowJo (BD Bioscience, Franklin Lakes, New Jersey). The gating strategies are given in Supplementary Figs. S1 and S2.

### Detection of covalently closed circular DNA (cccDNA)

Protein-free extrachromosomal DNA was isolated from 100,000 PMH cultured in 24-well format, using Hirt procedure. Remaining relaxed circular HBV DNA was digested with T5 exonuclease and quantitative PCR was performed to detect HBV as previously described^53^.

### Statistical analysis

Data are expressed as mean ± SD (standard deviation). Statistical significance was set at the level of *p* < 0.05. Unpaired t-test was performed as indicated.

### Institutional review board statement

The animal breeding scheme (Az. 81-02.04.2019.A344) and application experiments (Az. 84-02.04.2016.A126) were approved by the local committee Landesamt für Natur, Umwelt und Verbraucherschutz, LANUV.

### Supplementary Information

Supplementary Information.

## Data Availability

All the data generated during the current study are included in the manuscript.

## Abbreviations

ALT: Alanine aminotransferase
AST: Aspartate aminotransferase
BSA: Bovine serum albumin
cccDNA: Covalently closed circular DNA
ER: Endoplasmic reticulum
HBcAg: Hepatitis B core antigen
HBeAg: Hepatitis B extracellular antigen
HBsAg: Hepatitis B surface antigen
HBV: Hepatitis B virus
HBx: Hepatitis B × protein
HCC: Hepatocellular carcinoma
KC: Kupffer cells
LDH: Lactate dehydrogenase
LSEC: Liver sinusoidal endothelial cells
MC: Methylcellulose
PB: Phosphate buffer
PMH: Primary mouse hepatocytes
rNPC: Remaining non-parenchymal cells
RT: Room temperature
RT-PCR: Reverse transcription polymerase chain reaction
SD: Standard deviation
TEM: Transmission electron microscopy
UA: Uranyl acetate

## References

[1] GBDHepB & Collaborators (2022). Global, regional, and national burden of hepatitis B, 1990–2019: A systematic analysis for the Global Burden of Disease Study 2019. Lancet Gastroenterol. Hepatol..

[2] Alberts CJ (2022). Worldwide prevalence of hepatitis B virus and hepatitis C virus among patients with cirrhosis at country, region, and global levels: A systematic review. Lancet Gastroenterol. Hepatol..

[3] EASL & Collaborators (2017). EASL 2017 clinical practice guidelines on the management of hepatitis B virus infection. J. Hepatol..

[4] Suslov A, Boldanova T, Wang X, Wieland S, Heim MH (2018). Hepatitis B virus does not interfere with innate immune responses in the human liver. Gastroenterology.

[5] Bertoletti A, Ferrari C (2012). Innate and adaptive immune responses in chronic hepatitis B virus infections: Towards restoration of immune control of viral infection. Gut.

[6] Guidotti LG (1999). Viral clearance without destruction of infected cells during acute HBV infection. Science.

[7] Dupinay T (2013). Discovery of naturally occurring transmissible chronic hepatitis B virus infection among Macaca fascicularis from Mauritius Island. Hepatology.

[8] Walter E, Keist R, Niederost B, Pult I, Blum HE (1996). Hepatitis B virus infection of tupaia hepatocytes in vitro and in vivo. Hepatology.

[9] Roggendorf M, Kosinska AD, Liu J, Lu M (2015). The woodchuck, a nonprimate model for immunopathogenesis and therapeutic immunomodulation in chronic hepatitis B virus infection. Cold Spring Harb. Perspect Med..

[10] D'Ugo E (2010). The woodchuck hepatitis B virus infection model for the evaluation of HBV therapies and vaccine therapies. Expert Opin. Drug Discov..

[11] Reaiche GY, Le Mire MF, Mason WS, Jilbert AR (2010). The persistence in the liver of residual duck hepatitis B virus covalently closed circular DNA is not dependent upon new viral DNA synthesis. Virology.

[12] Li F, Wang Z, Hu F, Su L (2020). Cell culture models and animal models for HBV study. Adv. Exp. Med. Biol..

[13] Guidotti LG, Matzke B, Schaller H, Chisari FV (1995). High-level hepatitis B virus replication in transgenic mice. J. Virol..

[14] Halverscheid L (2008). Transgenic mice replicating hepatitis B virus but lacking expression of the major HBsAg. J. Med. Virol..

[15] Li H (2014). HBV life cycle is restricted in mouse hepatocytes expressing human NTCP. Cell Mol. Immunol..

[16] Belloni L (2012). IFN-α inhibits HBV transcription and replication in cell culture and in humanized mice by targeting the epigenetic regulation of the nuclear cccDNA minichromosome. J. Clin. Invest..

[17] Uprichard SL, Boyd B, Althage A, Chisari FV (2005). Clearance of hepatitis B virus from the liver of transgenic mice by short hairpin RNAs. Proc. Natl. Acad. Sci. USA.

[18] Chisari FV (1985). A transgenic mouse model of the chronic hepatitis B surface antigen carrier state. Science.

[19] Chisari FV (1989). Molecular pathogenesis of hepatocellular carcinoma in hepatitis B virus transgenic mice. Cell.

[20] Chisari FV (1987). Structural and pathological effects of synthesis of hepatitis B virus large envelope polypeptide in transgenic mice. Proc. Natl. Acad. Sci. USA.

[21] Real CI (2016). Hepatitis B virus genome replication triggers toll-like receptor 3-dependent interferon responses in the absence of hepatitis B surface antigen. Sci. Rep..

[22] Reifenberg K (1997). Long-term expression of the hepatitis B virus core-e- and X-proteins does not cause pathologic changes in transgenic mice. J. Hepatol..

[23] Schefczyk S (2023). Poly(I:C) induces distinct liver cell type-specific responses in hepatitis B virus-transgenic mice in vitro, but fails to induce these signals in vivo. Viruses.

[24] Ortega-Prieto AM, Cherry C, Gunn H, Dorner M (2019). In vivo model systems for hepatitis B virus research. ACS Infect. Dis..

[25] Prange R (2022). Hepatitis B virus movement through the hepatocyte: An update. Biol. Cell.

[26] Juelke K (2010). CD62L expression identifies a unique subset of polyfunctional CD56dim NK cells. Blood.

[27] Ndhlovu LC (2012). Tim-3 marks human natural killer cell maturation and suppresses cell-mediated cytotoxicity. Blood.

[28] Hood SP (2018). Phenotype and function of activated natural killer cells from patients with prostate cancer: Patient-dependent responses to priming and IL-2 activation. Front. Immunol..

[29] Freud AG (2013). Expression of the activating receptor, NKp46 (CD335), in human natural killer and T-cell neoplasia. Am. J. Clin. Pathol..

[30] Paolini R, Bernardini G, Molfetta R, Santoni A (2015). NK cells and interferons. Cytokine Growth Factor Rev..

[31] Quatrini L (2020). The immune checkpoint PD-1 in natural killer cells: Expression, function and targeting in tumour immunotherapy. Cancers.

[32] Iannacone M, Guidotti LG (2015). Mouse models of hepatitis b virus pathogenesis. Cold Spring Harb. Perspect. Med..

[33] Bazinet M (2022). HBsAg isoform dynamics during NAP-based therapy of HBeAg-negative chronic HBV and HBV/HDV infection. Hepatol. Commun..

[34] Chua PK, Tang FM, Huang JY, Suen CS, Shih C (2010). Testing the balanced electrostatic interaction hypothesis of hepatitis B virus DNA synthesis by using an in vivo charge rebalance approach. J. Virol..

[35] Li HC (2010). Nuclear export and import of human hepatitis B virus capsid protein and particles. PLoS Pathog..

[36] Michalak T, Nowoslawski A (1982). Crystalline aggregates of hepatitis B core particles in cytoplasm of hepatocytes. Intervirology.

[37] Naoumov NV (1993). Identification of hepatitis B virus-DNA in the liver by in situ hybridization using a biotinylated probe: Relation to HBcAg expression and histology. J. Hepatol..

[38] Chu CM, Yeh CT, Sheen IS, Liaw YF (1995). Subcellular localization of hepatitis B core antigen in relation to hepatocyte regeneration in chronic hepatitis B. Gastroenterology.

[39] Yoo JY (1990). Significance of hepatitis B core antigen in the liver in patients with chronic hepatitis B and its relation to hepatitis B virus DNA. J. Gastroenterol. Hepatol..

[40] Chen Y, Tian Z (2019). HBV-induced immune imbalance in the development of HCC. Front. Immunol..

[41] Kmiec Z (2001). Cooperation of liver cells in health and disease. Adv. Anat. Embryol. Cell Biol..

[42] Xu R, Huang H, Zhang Z, Wang FS (2014). The role of neutrophils in the development of liver diseases. Cell Mol. Immunol..

[43] Abel AM, Yang C, Thakar MS, Malarkannan S (2018). Natural killer cells: Development, maturation, and clinical utilization. Front. Immunol..

[44] Tian Z, Chen Y, Gao B (2013). Natural killer cells in liver disease. Hepatology.

[45] Bendelac A, Savage PB, Teyton L (2007). The biology of NKT cells. Annu. Rev. Immunol..

[46] Marotel M (2021). Peripheral natural killer cells in chronic hepatitis B patients display multiple molecular features of T cell exhaustion. Elife.

[47] Deerinck, T. J., Bushong, E. A., Thor, A. & Ellisman, M. H. *NCMIR methods for 3D EM: A new protocol for preparation of biological specimens for serial block face scanning electron microscopy*. (2010).

[48] Griffith J, Penalva MA, Reggiori F (2011). Adaptation of the Tokuyasu method for the ultrastructural study and immunogold labelling of filamentous fungi. J. Electron Microsc..

[49] Tokuyasu KT (1973). A technique for ultracryotomy of cell suspensions and tissues. J. Cell Biol..

[50] Schindelin J (2012). Fiji: An open-source platform for biological-image analysis. Nat. Methods.

[51] Liu J (2017). Advanced method for isolation of mouse hepatocytes, liver sinusoidal endothelial cells, and Kupffer cells. Methods Mol. Biol..

[52] Luo X (2021). Hippo pathway counter-regulates innate immunity in hepatitis B virus infection. Front. Immunol..

[53] Xia Y, Stadler D, Ko C, Protzer U (2017). Analyses of HBV cccDNA quantification and modification. Methods Mol. Biol..

