# Supplementary material for: Tg1.4HBV-s-rec mice, a crossbred hepatitis B virus-transgenic model, develop mild hepatitis
Source: Sci Rep. 2023 Dec 20;13:22829. doi: 10.1038/s41598-023-50090-8 (PMC10739827; doi:10.1038/s41598-023-50090-8)

Tg1.4HBV-s-rec mice, a crossbred hepatitis B virus-transgenic model, develop mild hepatitis

Stefan Schefczyk[1], Xufeng Luo[1,2], Yaojie Liang[1], Mike Hasenberg[3], Bernd Walkenfort[3], Martin Trippler[1], Jonas Schuhenn[4], Kathrin Sutter[4], Mengji Lu[4], Heiner Wedemeyer[1,5], Hartmut H. Schmidt[1], Ruth Broering[1]

1 Department of Gastroenterology, Hepatology and Transplant Medicine, Medical Faculty, University of Duisburg-Essen, Essen, Germany

2 Institute for Lymphoma Research, The Affiliated Cancer Hospital of Zhengzhou University & Henan Cancer Hospital, Zhengzhou, China

3 Electron Microscopy Unit, Imaging Center Essen, Medical Faculty, University of Duisburg-Essen, Germany Institute for Virology, University Hospital Essen, University of Duisburg-Essen, Essen, Germany

4 Institute for Virology, University Hospital Essen, University of Duisburg-Essen, Essen, Germany

5 Department of Gastroenterology, Hepatology and Endocrinology, Hannover Medical School, Hannover, Germany

Content

Supplementary Figures.. 2

Supplementary Tables.. 7

Original Western Blots.. 11

Supplementary Figures

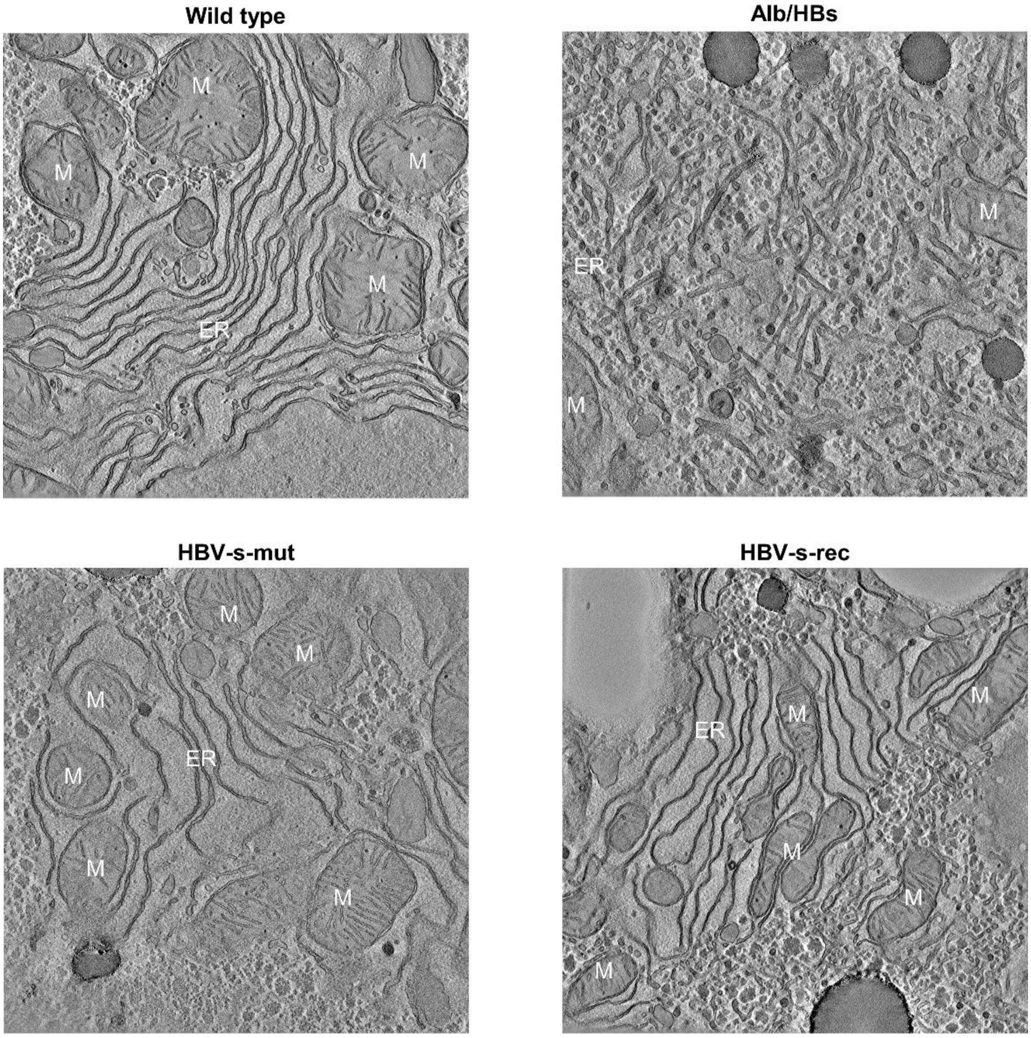

Figure S1. Endoplasmic reticulum malformations in Alb/HBs hepatocytes. (a) Liver tissue from 6-month-old wild type and HBV-transgenic mice (Alb/HBs, HBV-s-mut and HBV-s-rec) were fixated and contrasted according to a modified OTO protocol, with 250 nm tissue section thickness. Images were acquired using a JEM 1,400Plus TEM (JEOL Ltd, Tokyo, Japan) at 12,000× magnification. Images are representatives of each mouse strain (group sizes n = 3). ER, endoplasmic reticulum; M, mitochondria.

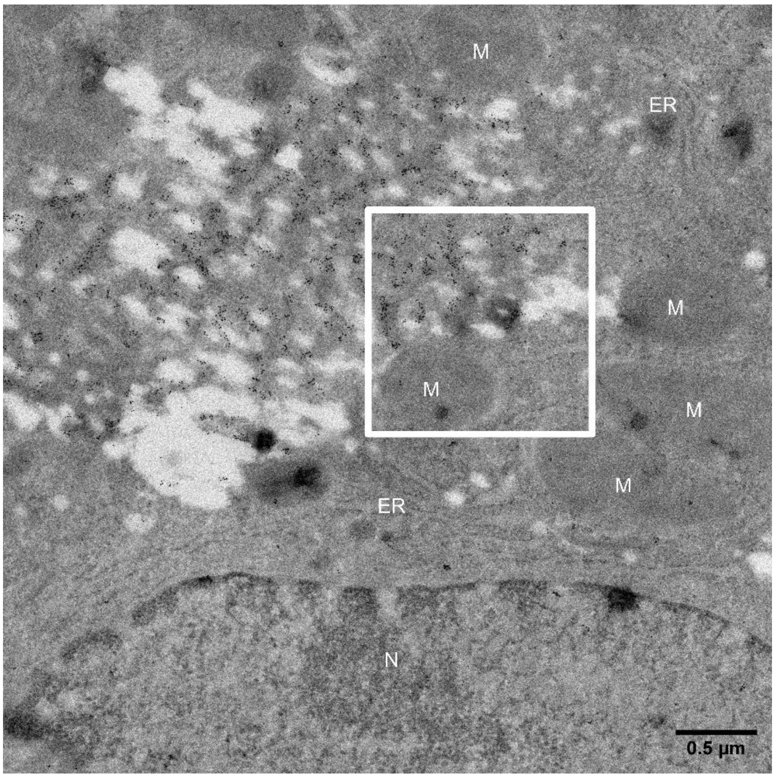

Figure S2. Visualising HBsAg in Alb/HBs strain. Liver tissue from 6-month-old Alb/HBs mouse were fixated and contrasted according to an immunogold staining protocol. Image is representative for the Alb/HBs strain (group size n = 3). White square indicates region of interest for HBsAg along endoplasmic reticulum extensions in Alb/HBs mouse. Scale bar, 0.5 µm. µm, micrometer; ER, endoplasmic reticulum; M, mitochondria; N, nucleus.

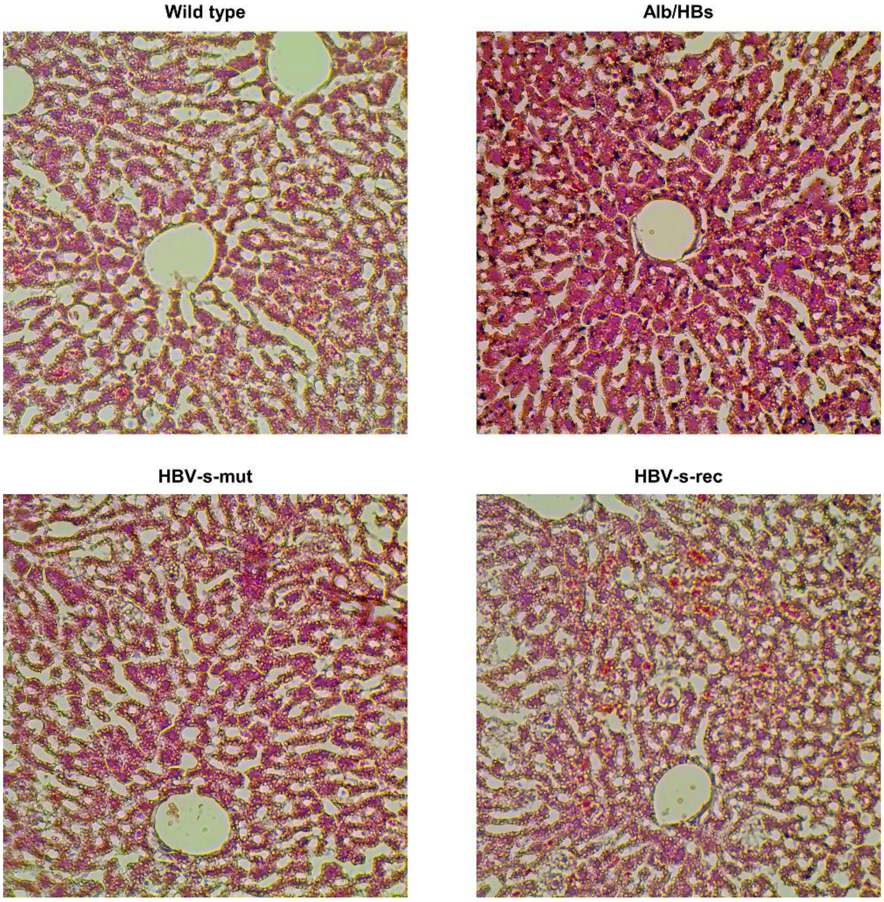

Figure S3. Hematoxylin and eosin staining in wild type and HBV-transgenic liver tissue. Liver cryosections from 3-month-old wild type and HBV-transgenic mice (Alb/HBs, HBV-s-mut and HBV-s-rec) were stained with hematoxylin and eosin staining. Images were taken using a Leica ICC50 HD microscope (Leica Microsystems, Wetzlar, Germany) with a 20× objective. Images are representatives of each mouse strain (group sizes n = 3).

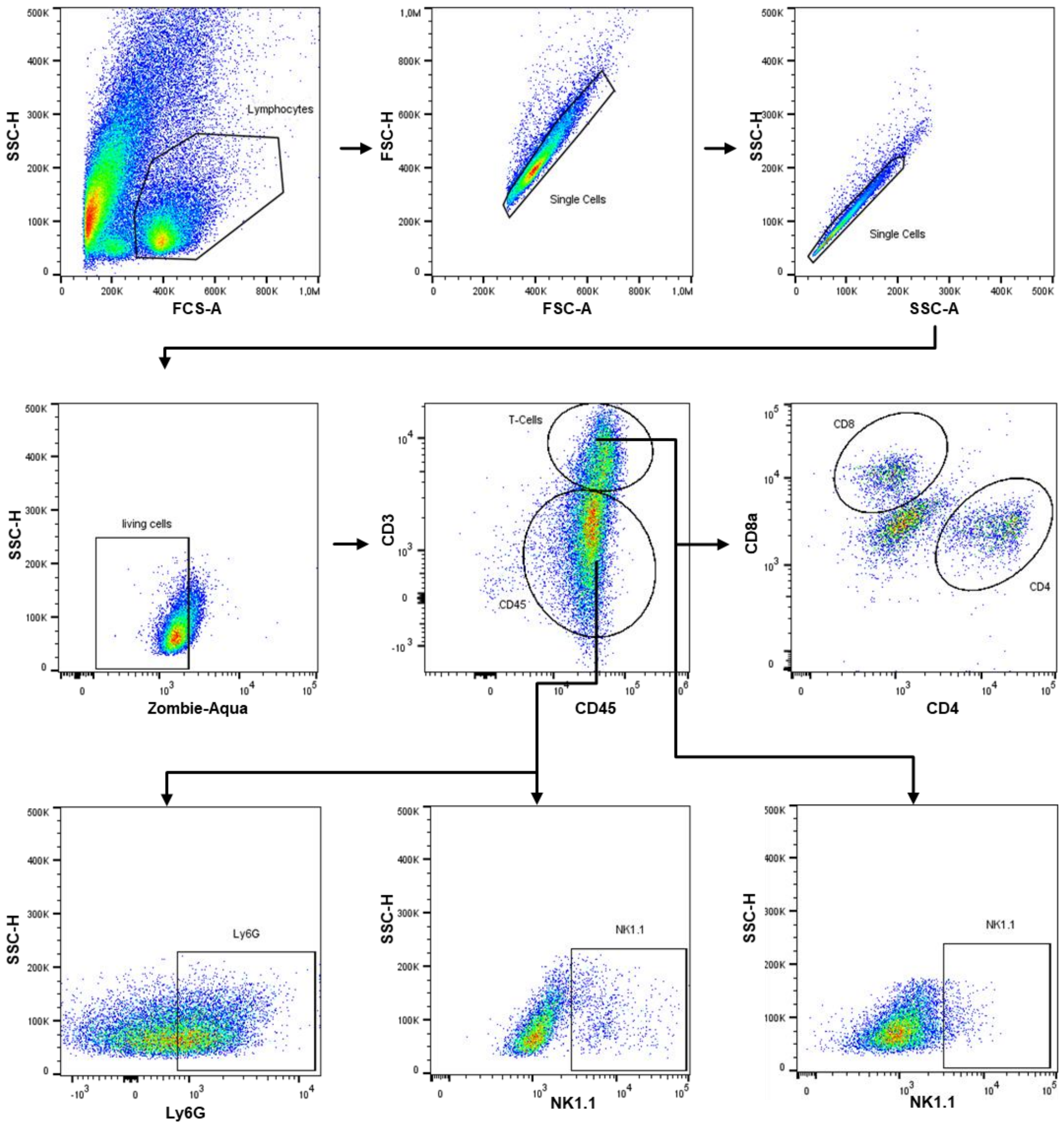

Figure S4. Gating strategy for rNPC flow cytometry. Gating the cells of interest was followed by double doublet exclusion and dead cell exclusion. Afterwards CD3⁺ and CD45⁺ cells were gated. CD45⁺ cells were further gated for Ly6G and NK1.1. CD3⁺ and CD45⁺ cells were further gated for CD4 and CD8a positivity. Abscissa and ordinate of the plots show the various flow cytometric channels. Flow cytometry was performed using the CytoFLEX S (Beckmann Coulter, Brea, California) and analysed using FlowJo (BD Bioscience, Franklin Lakes, New Jersey).

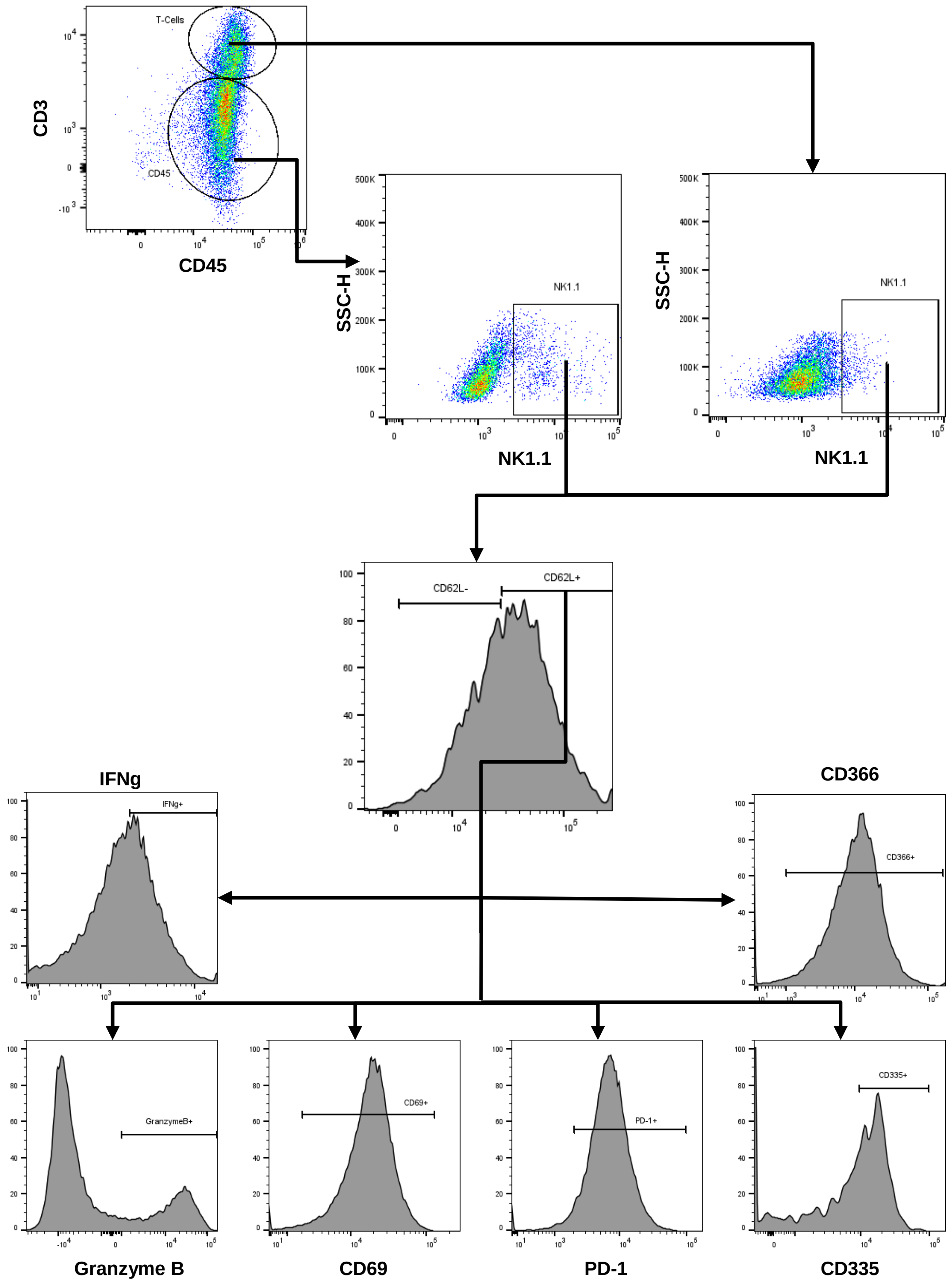

Figure S5. Gating strategy for NK and NKT cell marker flow cytometry. Gating the cells of interest was followed by double doublet exclusion and dead cell exclusion. Afterwards CD3+ and CD45+ cells were gated. CD45+ cells were further gated for NK1.1. CD3+ and CD45+ cells were further gated for NK1.1 positivity. Both NK and NKT cell populations were additionally gated for CD62L positivity. The CD62L-positive cells were subsequently gated for IFNg, GranzymeB, CD69, PD-1, CD335 and CD366. Abscissa and ordinate of the plots show the various flow cytometric channels. Flow cytometry was performed using the CytoFLEX S (Beckmann Coulter, Brea, California) and analysed using FlowJo (BD Bioscience, Franklin Lakes, New Jersey).

Supplementary Tables

Table S1. Primer sequences

**Target**	**Sense sequence**	**Antisense sequence**
Gapdh	5′-AAATTCAACGGCACAGTCAA-3′	5′-TCTCCATGGTGGTGAAGACA-3′
HBsAg	5′-CAGTGGAATTCCACAACC-3′	5′-AGAAAAACCCCGCCTGTA-3′
pregenomic RNA/ polymerase	5′-GCTTACAGACCACCAAATGC-3′	5′-TTCCCACCTTATGAGTCCAA-3′
HBxAg	5′-CCGTCTGTGCCTTCTCATCT-3′	5′-TAATCTCCTCCCCCAACTCC-3′

Table S2. Quantitative PCR failed to measure robust HBV cccDNA in hepatocytes

Sample Name	Target Name	CT	Calculated Quantity
NTC	HBV	35.60	0
Standard 1 (LOD)	HBV	32.35	1,000
Standard 2	HBV	27.72	10,000
Standard 3	HBV	24.09	100,000
Standard 4	HBV	20.76	1,000,000
HBV-s-mut 1	HBV	35.68	108.91
HBV-s-mut 2	HBV	34.82	185.35
HBV-s-mut 3	HBV	35.99	91.05
HBV-s-mut 4	HBV	33.59	411.80
HBV-s-rec 1	HBV	35.50	137.53
HBV-s-rec 2	HBV	34.97	167.18
HBV-s-rec 3	HBV	34.58	215.76
HBV-s-rec 4	HBV	33.31	452.72

LOD, limit of detection; NTC, non-template control

Table S3. Protocol for microwave-assisted chemically processed mouse liver tissue

Step	Chemical	Repetition	Time	Power (W)	Temp. (°C)	Vacuum
1	PHEM	4×	40s	250	20	Off
2	1% OS + 1,5% PFC in 1x PHEM	10 cycles microwaves on-off	2min	100	20	On
3	PHEM	2×	40s	250	20	Off
4	H_2O	2×	40s	250	20	Off
5	1% TCH	8 cycles microwaves on-off	2min per cycle	100	20	On
6	H_2O	4×	40s	250	20	Off
7	1% OS in H_2O	10 cycles microwaves on-off	2min per cycle	100	20	On
8	H_2O	4×	40s	250	20	Off
9	1% UA in H_2O	10 cycles microwaves on-off	2min per cycle	100	20	On
10	H_2O	4×	40s	250	20	Off
11	Dehydration 30%, 50%, 70%, 80%, 90%, 3x 100% EtOH, 2x aceton		40s	250	20	Off
12	Infiltration EPON in aceton 1:3, 1:1, 3:1, 3x pure		3min	250	20	On

EPON, Poly/Bed® 812; EtOH, ethanol; min, minutes; OS, osmium, osmium tetroxide; PFC, potassium ferrocyanide; s, seconds; TCH, thiocarbohydrazide; UA, uranyl acetate

Table S4. Primary antibodies and immunogold reagents for immunogold staining

Antibody	Company	Order No.	Concentration
HBsAg	Aviva Systems Biology	OAMA02794	10 ng/ml
HBcAg	Abcam	AB8637	10 ng/ml
Goat-anti-Mouse IgG (H&L)	Aurion	#806.022	1 : 20
Goat-anti-Rabbit IgG (H&L)	Aurion	#806.011	1 : 20

Table S5. Western blot primary and secondary antibodies

Antibody	Company	Order No.	Concentration
HBsAg	Aviva Systems Biology	OAMA02794	1 : 500
HBcAg	Abcam	AB8637	1 : 500
GAPDH (D4C6R)	Cell signaling	97166S	1 :1,000
Anti-rabbit IgG, HRP-linked Antibody	Cell signaling	7074S	1 : 5000
Anti-mouse IgG, HRP-linked Antibody	Cell signaling	7076S	1 : 2000

GAPDH, Glyceraldehyde 3-phosphate dehydrogenase; HBsAg, Hepatitis B surface antigen; HBcAg, Hepatitis B core antigen; HRP, horseradish peroxidase.

Table S6. Immunofluorescence primary and secondary antibodies

Antibody	Company	Order No.	Concentration
HBsAg	Aviva Systems Biology	OAMA02794	1 : 500
HBcAg	Abcam	AB8637	1 : 100
HBxAg	BioVendor	RD981038100	1 : 100
Donkey anti-mouset IgG, Alexa Fluor™ 488	Invitrogen	A21202	1 : 500
Donkey anti-rabbit IgG, Alexa Fluor™ Plus 680	Invitrogen	A32802	1 : 500
Alexa Fluor® 594 AffiniPure Goat Anti-Horse IgG (H+L)	Jackson ImmunoResearch Europe LTD.	108-585-003	1 : 200

Table S7. FACS antibodies

Antibody	Fluorochrome	Company	Order No.	Dilution
CD3	APC/Fire 750	Biolegend	152308	0.2µg/ml
CD4	FITC	Biolegend	100510	0.5µg/ml
CD45	Alexa Fluor 700	Biolegend	147716	0.5µg/ml
CD62L	PE/Cy7	Biolegend	104418	0.2µg/ml
CD8a	Per/CP/Cyanine5.5	Biolegend	100733	0.2µg/ml
FAS	Brilliant Violet 605	Biolegend	152612	0.2µg/ml
FASL	APC	Biolegend	106610	0.2µg/ml
Ly6G	AF594	Biolegend	127636	0.5µg/ml
NK1.1	Brilliant Violet 421	Biolegend	108741	0.2µg/ml
Zombie Aqua	Brilliant Violet 510	Biolegend	423102	1 : 1000

Original Western Blots

Figure 2b

HBsAg

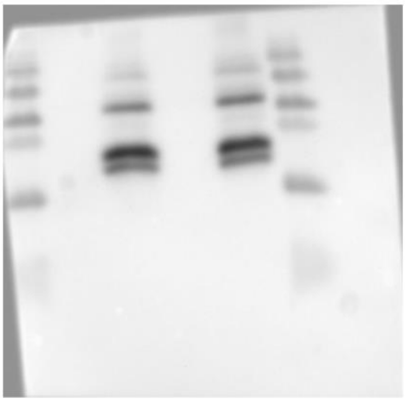

HBcAg

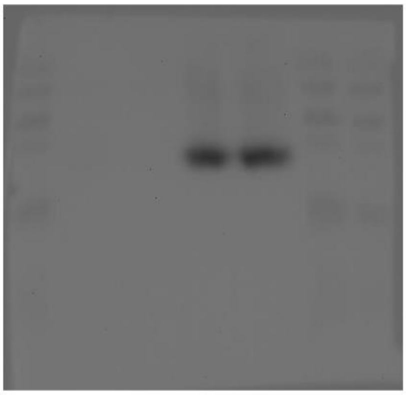

Actin (reused blot)

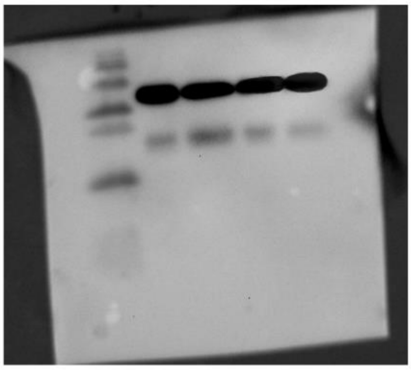

Supplement: Supplementary file 1 — Supplementary Information. [file 41598_2023_50090_MOESM1_ESM.pdf]
